# Severe Respiratory Syncytial Virus Infection

**DOI:** 10.1007/s12098-019-03118-9

**Published:** 2019-12-21

**Authors:** Yasuyo Kashiwagi, Toshihiro Nakayama, Masahiro Kimura, Tomoko Maeda, Soken Go, Hisashi Kawashima, Akihito Sawada, Tetsuo Nakayama

**Affiliations:** 1 Department of Pediatrics and Adolescent Medicine, Tokyo Medical University, 6-7-1 Nishishinjuku, Shinjuku-ku, Tokyo, 160-0023 Japan; 2 Laboratory of Viral Infection, Kitasato Institute for Life Sciences, Kitasato University, Tokyo, Japan

*To the Editor:* We previously reported a case of twin neonates with severe respiratory syncytial virus (RSV) infection [1]. Here we describe a 26-d-old boy with severe RSV infection and a 16-mo-old boy with RSV infection who died suddenly.

The first case is a 26-d-old boy who was born at 36 wk and 4 d of gestation. His birth weight was 2874 g, and he showed respiratory distress with left pneumothorax and was intubated for 8 d. He developed coughing and sneezing on day 25 and was admitted to our ward on day 26. The RSV rapid assay, based on immunochromatography with nasal fluid (Check RSV; Alfresa, Japan) was positive. His severe respiratory distress required mechanical ventilation from day 27 to day 41.

The second case is a 16-mo-old boy who was completely healthy until this episode. He was coughing and sneezing for several days before his sudden death. He had a high fever and developed convulsions for 1 min and was found in cardiopulmonary arrest an hour later. He was referred to our emergency room, but resuscitation was unsuccessful. The result of the RSV rapid assay using nasal fluid was positive.

The patients’ nasopharyngeal aspirate samples obtained in the acute phase were analyzed by real-time RT-PCR [2], which showed high levels of RSV type B, at 4.4 × 10^5^ and 2.4 × 10^3^ copies/μg viral RNA in the first and second patient, respectively.

We measured the viral load in 36 children with not severe RSV infection. The average load were 4.5 × 10^4^ copies/μg viral RNA (data not shown). In our present cases, the amount of RSV found in the neonate was higher than that that in the young child with sudden death.

Kakimoto et al. demonstrated that an extreme elevation of IL-6 might predict the risk for sudden death in normally developed children with RSV infection [3]. However, the mechanism of rapid progression of RSV-induced sudden death remains to be elucidated.

Regardless of the amount of RSV, unexpected complications such as central nervous system infection and dysfunction of the host immune system may happen in a fatal case [4].
